# Supplementary material for: Hypoxia controls plasma membrane targeting of polarity proteins by dynamic turnover of PI4P and PI(4,5)P2
Source: eLife. 2022 Jun 9;11:e79582. doi: 10.7554/eLife.79582 (PMC9242647; doi:10.7554/eLife.79582)
Supplement: Supplementary file 2. [file elife-79582-supp2.docx]

**Primers used in this study:**

**Primers for generating pNP-PI4KIIIα-KD**

5’-ctagcagtCCCGTTCTCAAGAGTAGCAAAtagttatattcaagcataTTTGCTACTCTTGAGAACGGGgcg-3’

5’-aattcgcCCCGTTCTCAAGAGTAGCAAAtatgcttgaatataactaTTTGCTACTCTTGAGAACGGGactg-3’

**Primers for generating pNP-PI4KIIα-KD**

5’-ctagcagtATGGTCATATTCAATAATTAAtagttatattcaagcataTTAATTATTGAATATGACCATgcg-3’

5’-aattcgcATGGTCATATTCAATAATTAAtatgcttgaatataactaTTAATTATTGAATATGACCATactg-3’

**Primers for generating pNP-fwd-KD**

5’-ctagcagtACGGTTATCAATACTACACTAtagttatattcaagcataTAGTGTAGTATTGATAACCGTgcg-3’

5’-aattcgcACGGTTATCAATACTACACTAtatgcttgaatataactaTAGTGTAGTATTGATAACCGTactg

**Primer for generating pGU-P4M::GFP**

5’- tcgGCTAGCggctagcgaacaggcATGacggcaagcacggaaaactttaaaaatgTTAAAGAAAAATATCAGTGTCGGCGCGCC TTTTATCTTAATGGTTTGTCTTTCTTG

5’- AGTGTCGGCGCGCC TTTTATCTTAATGGTTTGTCTTTCTTG

**Primer for generating pGU-P4MX2::GFP**

5’-AGTGTCGCTAGCggctagcgaacaggcATGGTGAGCAAGGGCGAGGAGCTGTTCAC

5’-CTGGACACGCGTTTATGATCAGTTATCTAGATCCGGTGGA

**Primer for generating pGU-PLC-PH::GFP**

5’-tcgAGATCT ggc tag cga aca ggc ATG ATGGACTCGGGCCGGGACTTC

WD417: AGTGTC GGTACC TTA GTTCAGATCCTCCTCGGA

**Primer for generating pGU-PLC-PH::RFP**

5’-tcgAGATCTggctagcgaacaggcATGATGGACTCGGGCCGGGACTTC

5’-AAGGAGCTGCAGAACTTCCTGAAGGTGTCTAAGGGCGAAGAGCTGATTAAG

5’-CTTAATCAGCTCTTCGCCCTTAGACACCTTCAGGAAGTTCTGCAGCTCCTT

5’-AGTGTCGGTACCTTAATTAAGTTTGTGCCCCAGTTT
